# Baseline NT-proBNP nonresponse score and health status measures in assessing treatment responses in heart failure with reduced ejection fraction

**DOI:** 10.1016/j.ahj.2025.01.011

**Published:** 2025-01-25

**Authors:** Thanat Chaikijurajai, Horng H. Chen, W.H. Wilson Tang

**Affiliations:** a Department of Cardiovascular Medicine, Mayo Clinic, Rochester, MN; b Kaufman Center for Heart Failure Treatment and Recovery, Heart Vascular and Thoracic Institute, Cleveland Clinic, Cleveland, OH; c Cleveland Clinic Lerner College of Medicine of Case Western Reserve University, Cleveland, OH.

## Abstract

**Background:**

We aim to validate NT-proBNP nonresponse score (NNRS) previously derived from the PROTECT and BATTLESCARRED studies in comparison with standard health status measures in predicting natriuretic peptide responses in patients with heart failure with reduced ejection fraction.

**Methods:**

Data on the GUIDE-IT trial were used to derive the NNRS based on 4 predictors including baseline NT-proBNP, heart rate, NYHA functional class, and history of atrial fibrillation. The discriminative capacity of the NNRS and health status measures for having NT-proBNP >1,000 pg/mL at 12 months was assessed and compared with baseline or follow-up health status measures including Kansas City Cardiomyopathy Questionnaire Overall Summary Score (KCCQ-OSS), Duke Activity Status Index (DASI), and 6-minute walk distance. Multivariable logistic regression analysis was used to determine the predictive value of the score and health status measures greater than the median values for NT-proBNP response with adjustment for age, sex, body mass index, comorbidities, baseline creatinine and NT-proBNP levels.

**Results:**

Among 877 patients, 252 (28.7%) patients had NT-proBNP >1,000 pg/mL at 12 months. The discriminative capacity of the NT-proBNP nonresponse score was 0.72 (95% CI, 0.67–0.77). After adjusting for covariates, only NNRS (*P* = .044) and KCCQ-OSS (*P* = .002) remained predictive for NT-proBNP nonresponse at 12 months.

**Conclusion:**

NT-proBNP nonresponse score and KCCQ-OSS was associated with persistently elevated NT-proBNP 12 months independently of baseline NT-proBNP levels.

## Background

Although there has been consistent evidence suggesting that persistently elevated natriuretic peptide levels over time despite guideline directed medical therapy (GDMT) optimization are associated with poor prognosis,^1–4^ using natriuretic peptide-guided medical therapy in patients with heart failure (HF) with reduced ejection fraction (HFrEF) remains unclear and has not been consistently utilized in clinical practice.^5–10^

The Guiding Evidence Based Therapy Using Biomarker Intensified Treatment in Heart Failure (GUIDE-IT) ^10^ trial was the largest multicenter randomized controlled trial that randomized patients with chronic HFrEF to either NT-proBNP-guided HF therapy with NT-proBNP goal of less than 1,000 pg/mL or usual care. The study revealed that there was no statistically significant difference in cardiovascular (CV) mortality, HF hospitalization, GDMT optimization, or NT-proBNP levels between both groups. However, *posthoc* analyses from the GUIDE-IT study still demonstrate the association between achieving NT-proBNP target and improved outcomes.^4,11^

While most studies strive to identify treatment responders, identifying those that are unlikely to respond is as important as appropriate triage of case may be warranted. Simple clinical tools that can early identify patients with HFrEF who are likely to not respond to treatment are needed to help clinicians determine the frequency and interval for follow ups, GDMT titration and optimization, and/or prompting the need for advanced HF therapy evaluation. Over a decade ago, Gaggin et al.^12^ developed the NT-proBNP Non-Response Score (NNRS) for predicting future natriuretic peptide response. The risk score was developed from the cohort of 151 patients with HFrEF enrolled in the Use of Amino-Terminal Pro–B-Type Natriuretic Peptide to Guide Outpatient Therapy of Patients With Chronic Left Ventricular Systolic Dysfunction (PROTECT) trial,^8^ and validated in the cohort of 364 patients with HFrEF enrolled in the N-Terminal Pro–B-Type Natriuretic Peptide-Guided Treatment for Chronic Heart Failure: Results From the BATTLESCARRED (NT-proBNP–Assisted Treatment To Lessen Serial Cardiac Readmissions and Death) Trial.^9^ However, the NNRS has not been validated in a large cohort of patients with HFrEF with contemporary GDMT use.

On the other hand, baseline health status measures, such as Kansas City Cardiomyopathy Questionnaire (KCCQ), Duke Activity Status Index (DASI), and 6-minute walk distance (6MWD), have been shown to be associated with prognosis in patients with HFrEF.^13–18^ However, their abilities to identify those who are more likely to respond to GDMT optimization (as indicated by improvements in NT-proBNP) in addition to NT-proBNP or other clinical presentations as captured in NNRS have not been explored. Moreover, there has not been a comprehensive prognostic approach to predicting NT-proBNP response or prognosis that utilizes NT-proBNP levels, symptoms and health status measures. Herein, we sought to validate the prognostic value NNRS and health status measures (alone and in combination) in identifying those with NT-proBNP responses and more favorable clinical outcomes in patients with HFrEF enrolled in the GUIDE-IT trial.

## Methods

Details of the GUIDE-IT trial have been previously described elsewhere,^10^ and the trial dataset was obtained through the publicly available NIH Biologic Specimen and Data Repository Information Coordinating Center (BioLINCC). Briefly, GUIDE-IT was a multicenter, randomized controlled trial of 894 patients with chronic HFrEF (left ventricular ejection fraction [LVEF] ≤40%), a history of a HF event such as HF hospitalization, emergency department visit for HF, and outpatient treatment with intravenous diuretics for HF within the prior 12 months, and elevated natriuretic peptides within the prior 30 days. Patients were randomized in a 1:1 fashion to either NT-proBNP-guided strategy (446 patients) or usual care (448 patients) for GDMT optimization recommended by the standard HF guidelines at the time of the enrollment. The study protocol was approved at each participating institutional review board or ethics committee. All study participants provided written informed consent before enrollment. The current analysis was approved by the Institutional Review Board of Cleveland Clinic Foundation.

The NNRS composed of 4 predictors and their integeric score including baseline NT-proBNP (<1,000 pg/mL, 1,000–5,000 pg/mL, and >5,000 pg/mL; Score = 0, 2, 3, respectively), heart rate <60 beats per min (Score = 1), New York Heart Association (NYHA) functional class III or IV (Score = 1), and history of AF (Score = 1).^12^ The score ranges from 0 to 6 with 3 being the optimal cut-off for predicting NT-proBNP response at 10 months.^12^

Available baseline health status measures included the KCCQ Overall Summary Score (KCCQ-OSS), ^13,14^ DASI,^15,16^ and 6MWD.^17,18^ The KCCQ is a 23-item disease-specific instrument that maps to 7 domains such as symptom frequency, symptom burden, symptom stability, physical limitations, social limitations, quality of life and self-efficacy that ranges from 0 (worst) to 100 (best) .^13^ DASI is a simple 12-item instrument that was developed for assessment of cardiac-related functional capacity, and shown to correlate well with peak oxygen consumption that ranges from 0 (worst) to 58.2 (best).^15^ Six-minute walk test is a simple method to assess functional capacity and exercise tolerance, and has been recommended as 1 of the standard objective assessment of exercise capacity by the latest HF guidelines.^19^ It can be performed by having the patient walk for 6 min on a measured flat course with consistent timing of encouragement.^17^

The primary outcome of this *posthoc* analysis is NT-proBNP response, which was defined by demonstrating NT-proBNP level of <1,000 pg/mL at 12 months as part of the GUIDE-IT study. The secondary outcomes included NT-proBNP response at 6 months, as well as composite of CV mortality and HF hospitalizations.

Clinical characteristics are presented as median (interquartile rate [IQR]) and percentage (%) for continuous and categorical variables, respectively. Differences in baseline characteristics across NNRS tertiles were evaluated by the Kruskall-Wallis or Chi-square test for the continuous and categorical variables, respectively. Harrell’s C-statistics ^20^ were used to estimate the discriminative capacity of the NNRS, baseline NT-proBNP, KCCQ-OSS, DASI and 6MWD for NT-proBNP response at 6, 12 months and composite of CV mortality and HF hospitalization. The association between having NNRS above 3, health status measures less than the median values, and NT-proBNP response was performed using both univariable and multivariable logistic regression analysis with adjustment for age, sex, body mass index (BMI), comorbidities (ischemic heart disease, stroke, AF, hypertension, chronic obstructive pulmonary disease [COPD], diabetes mellitus [DM], dyslipidemia, and peripheral artery disease [PAD]), LVEF, baseline serum creatinine, and NT-proBNP. Scatter plots with Spearman’s correlation analysis were used to assess concordance and distribution among KCCQ-OSS, DASI and 6MWD. Since there are no standard cut-offs for these health status measures recommended by the contemporary HF guidelines, the median values were arbitrarily chosen to simplify their potential clinical application. Survival curves for NNRS tertiles, as well as numbers of abnormal values (less than median) combining NNRS and KCCQ-OSS were estimated using the Kaplan-Meier method, compared using the log-rank test and univariable and multivariable Cox regression analysis with adjustment for the previously mentioned covariates. In addition, baseline characteristics and survival of patients low and high NNRS at baseline and 1 year were also assessed. Correction for multiple comparison testing was not performed, and these analyses can be considered exploratory. Two-sided *P* values of less than .05 were considered statistically significant. All statistical analyses were performed using SPSS version 23 (IBM, Chicago IL).

## Results

### Baseline characteristics

In our study cohort (877 patients), we excluded a total of 17 patients who had missing data required for the NNRS calculation including 13 patients with missing baseline NYHA functional class, 7 patients with missing baseline heart rate, and 4 patients with missing both baseline NYHA functional class and heart rate. Baseline characteristics across NNRS tertiles of the study population are shown in Tab. 1. Among 877 patients included in the study, 314 patients had score of 0–2 (tertile 1), 275 patients had score of 3 (tertile 2), and 288 patients had score of 4–6 (tertile 3). Median of NNRS was 3 (IQR 2–4), while median KCCQ-OSS, DASI, and 6MWD were 59 (IQR 40–75), 7.3 (IQR 2.8–15.5), and 305 (IQR 202–375) meters, respectively. Patients with higher tertile of NNRS were likely to be older, had lower BMI, had greater burden of comorbidities, higher LVEF, NYHA functional class, baseline serum creatinine, NT-proBNP and digoxin use, but lower angiotensin converting enzyme inhibitor/angiotensin receptor blocker use, and worse health status measures (Tab. 1).

### Correlation between the NNRS and health status measures

**Figure 1.**
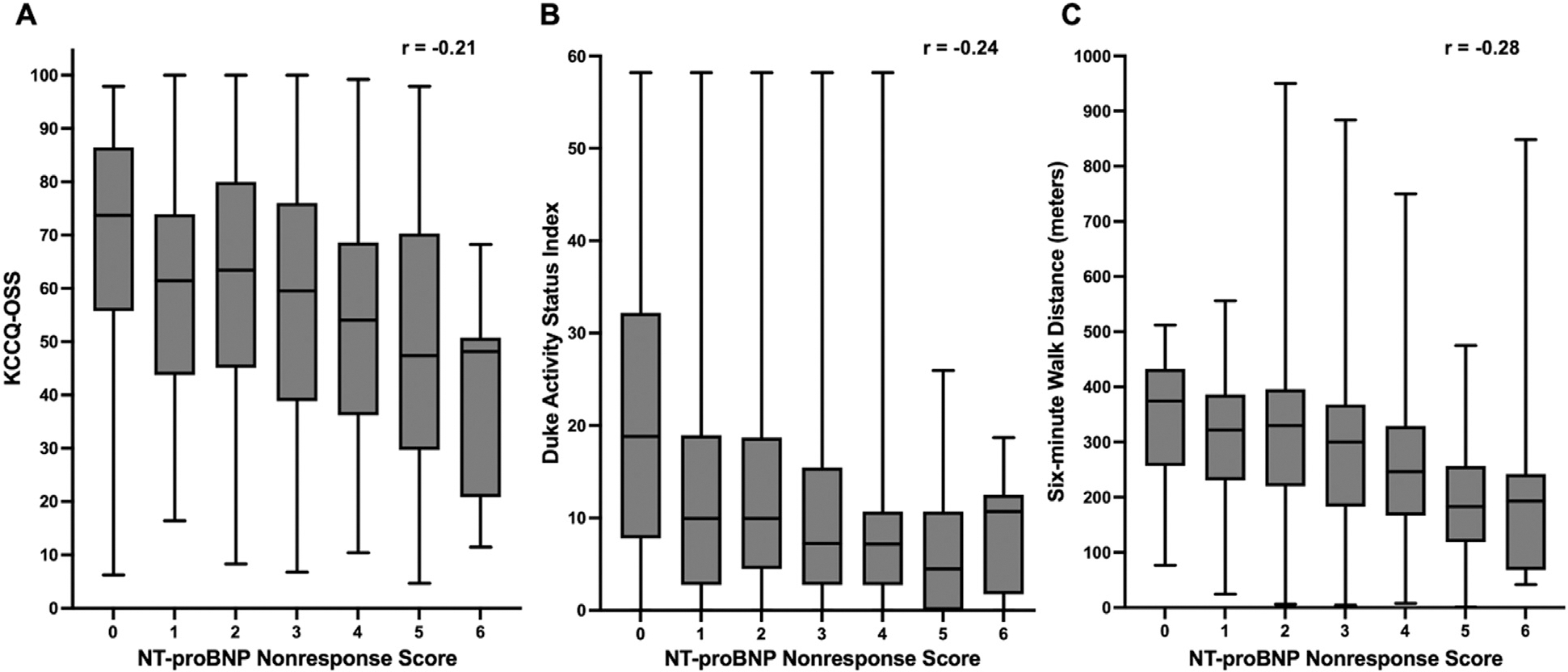
Box and whisker plots for the NT-proBNP nonresponse score and health status measures. The NT-proBNP nonresponse score correlated poorly with Kansas city cardiomyopathy questionnaire overall summary score (KCCQ-OSS) **A**, Duke activity status index **B**, and 6- minute walk distance **C**, with Spearman’s Correlation Coefficients (r) of −0.21, −0.24, −0.28, respectively.

Fig. 1 shows the Box and Whisker plot for the NNRS and KCCQ-OSS, DASI and 6MWD. NNRS correlated poorly with KCCQ-OSS (*r* = −0.21, Fig. 1A), DASI (*r* = −0.24, Fig. 1B) and 6MWD (*r* = −0.28, Fig. 1C). Scatter plots with median values and Spearman’s correlation coefficients between baseline NNRS with DASI, KCCQ-OSS and 6MWD are shown in Supplementary Figure S1. KCCQ-OSS correlated better with DASI (*r* = 0.56) than 6MWD (*r* = 0.31), while DASI and 6MWD also correlated relatively poorly (*r* = 0.36).

### Discriminative capacity of the NNRS, baseline NT-proBNP, and HEALTH status measures for NT-proBNP response and clinical outcomes

Among 592 and 443 patients who had NT-proBNP levels available at 6 and 12 months, respectively, there were 378 (63.9%) and 252 (56.9%) patients observed to have NT-proBNP level of >1,000 pg/mL at 6 and 12 months, respectively. Supplemental Table S1 shows C-statistics of the baseline NT-proBNP, NNRS, and health status measures associated with lack of NT-proBNP response at 6, 12 months and prognosis. For lack of NT-proBNP response at 6 months, baseline NT-proBNP levels had the highest C-statistic (0.78, 95% confidence interval [CI], 0.74–0.82) and closely followed by the NNRS (0.73, 95% CI, 0.69–0.78), while 6MWD (0.63, 95% CI, 0.58–0.67), KCCQ-OSS (0.56, 95% CI, 0.51–0.60) and DASI (0.54, 95% CI, 0.50–0.59) demonstrated lower C-statistics. These trends were similar at 12 months, with baseline NT-proBNP (0.75, 95% CI, 0.70–0.80) and NNRS (0.72, 95% CI, 0.67–0.77) having the highest C-statistic, followed by 6MWD (0.65, 95% CI, 0.60–0.70), KCCQ-OSS (0.59, 95% CI, 0.54–0.65) and DASI (0.56, 95% CI, 0.51–0.62), respectively.

During median follow up of 300 days, 321 (36.6%) patients had CV mortality or HF hospitalization (107 [12.2%] patients with CV mortality and 282 [32.2%] patients with HF hospitalization). The NNRS had the highest C-statistic (0.64, 95% CI, 0.60–0.67) followed by 6MWD (0.62, 95% CI, 0.58–0.66), baseline NT-proBNP levels (0.60, 95% CI, 0.57–0.64), KCCQ-OSS (0.60, 95% CI, 0.56–0.64), while DASI had the lowest (0.57, 95% CI, 0.53–0.60), respectively (Supplementary Table S1).

### Prognostic value of the NNRS and health status measures

Using the median value as a cut-off for each variable, higher NNRS and lower health status measures were associated with higher likelihood of lack of NT-proBNP response at 6 months and 12 months (Tab. 2). However, after adjusting potential confounders, only KCCQ-OSS (adjusted odds ratio [OR] 1.55, 95% CI, 1.03–2.32, *P* = .034) remained predictive of NT-proBNP response at 6 months. For 12 months, higher NNRS (adjusted OR 1.99, 95% CI, 1.02–3.90, *P* = .044) and KCCQ-OSS (adjusted OR 2.14, 95% CI, 1.33–3.45, *P* = .002) were independently associated with higher risk of lack of NT-proBNP response at 12 months (Tab. 3).

In the univariable logistic regression analysis using the median value as a cut-off for each variable, higher NNRS and lower health status measures were associated an increased risk of CV mortality and HF hospitalization (Tab. 2). In the multivariable model, the association between NNRS (adjusted OR 1.79, 95% CI, 1.36–2.36), KCCQ-OSS (adjusted OR 1.58, 95% CI, 1.25–1.99), DASI (adjusted OR 1.49, 95% CI, 1.18–1.88), 6MWD (adjusted OR 1.82, 95% CI, 1.37–2.42) and the composite of CV mortality and HF hospitalization remained robust (Tab. 3, all *P ≤* .001).

**Figure 2.**
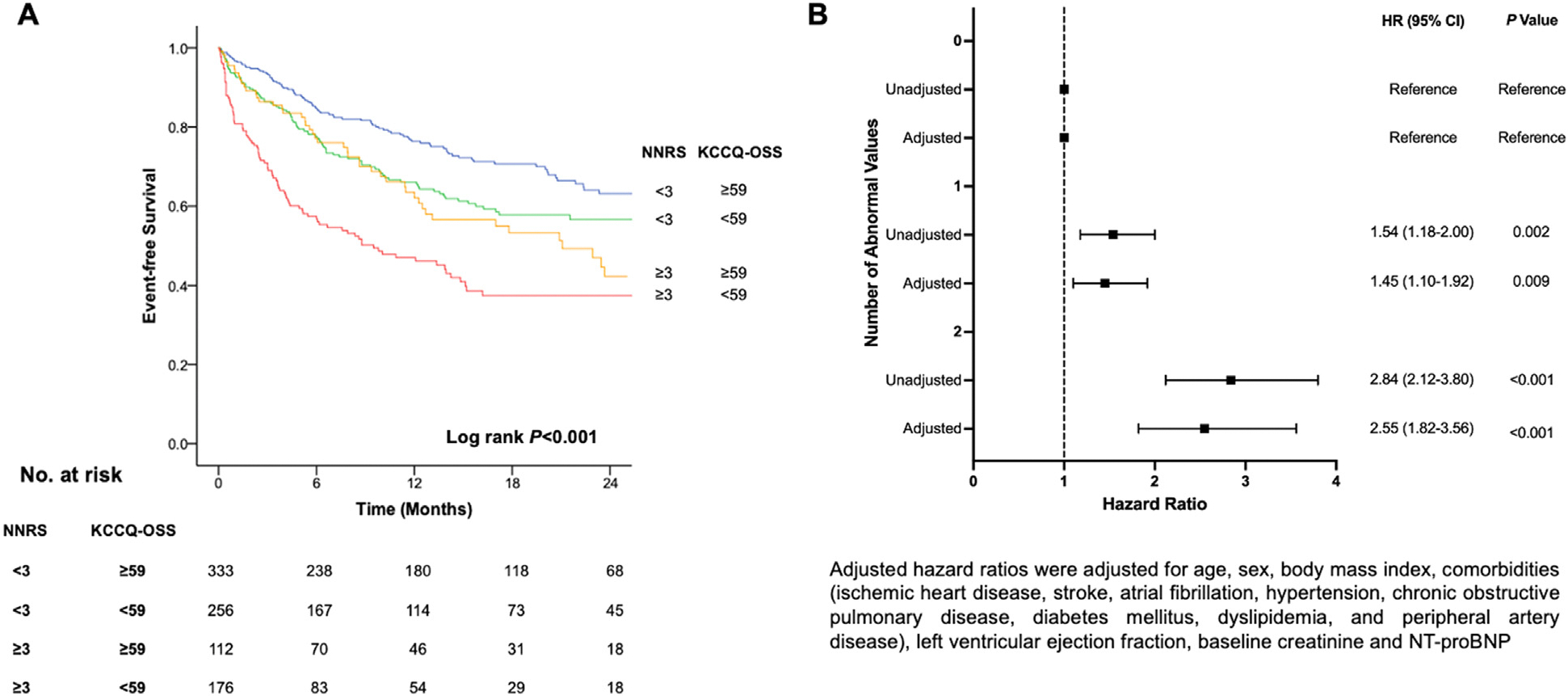
Prognostication of combined NT-proBNP nonresponse score and Kansas city cardiomyopathy questionnaire overall summary score. **A,** Using a cut-off value of 3 for the NT-proBNP nonresponse score (NNRS) and the median value of 59 for the Kansas City Cardiomyopathy Questionnaire overall summary score (KCCQ-OSS), patient with low NNRS but high KCCQ-OSS had the best survival from cardiovascular death and heart failure hospitalization, followed by patients with low NNRS and low KCCQ-OSS, patients with high NNRS and high KCCQ-OSS, and high NNRS and low KCCQ-OSS, respectively. **B,** Having 1 or 2 abnormal values of NNRS or KCCQ-OSS was associated with increased risk cardiovascular (CV) mortality and HF hospitalization independently of age, sex, BMI, comorbidities (ischemic heart disease, stroke, atrial fibrillation [AF], hypertension, chronic obstructive pulmonary disease [COPD], diabetes mellitus [DM], dyslipidemia, and peripheral artery disease [PAD]), left ventricular ejection fraction (LVEF), baseline serum creatinine, and NT-proBNP levels.

Kaplan-Meier curves for CV mortality and HF hospitalization across NNRS tertiles are illustrated in Supplementary Figure S2 (log rank *P* < .001). Given that the NNRS and KCCQ-OSS were the only 2 independent predictors of NT-proBNP response at 12 months, both the NNRS and KCCQ-OSS stratified patients into 4 groups based on their prognosis with low NNRS but high KCCQ-OSS being associated with the best prognosis followed by low NNRS and low KCCQ-OSS, high NNRS and high KCCQ-OSS, and high NNRS and low KCCQ-OSS, respectively (Fig. 2A, log rank *P* < .001). Using a cut-off value of 3 for the NNRS and the median value of 59 for the KCCQ-OSS, higher number of abnormal values of NNRS and KCCQ-OSS was significantly associated with decreased survival from CV mortality and HF hospitalization (Fig. 2B, 1 vs 0 abnormality, hazard ratio [HR] 1.54, 95% CI, 1.18–2.00; 2 vs 0 abnormalities, HR 2.84, 95% CI, 2.12–3.80). The association remained robust even after adjusting for after adjusting for age, sex, BMI, comorbidities, LVEF, baseline serum creatinine, and NT-proBNP (Fig. 2B, 1 vs 0 abnormality, adjusted HR 1.45, 95% CI, 1.82–3.56; 2 vs 0 abnormalities, adjusted HR 2.55, 95% CI, 1.92–3.56).

**Figure 3.**
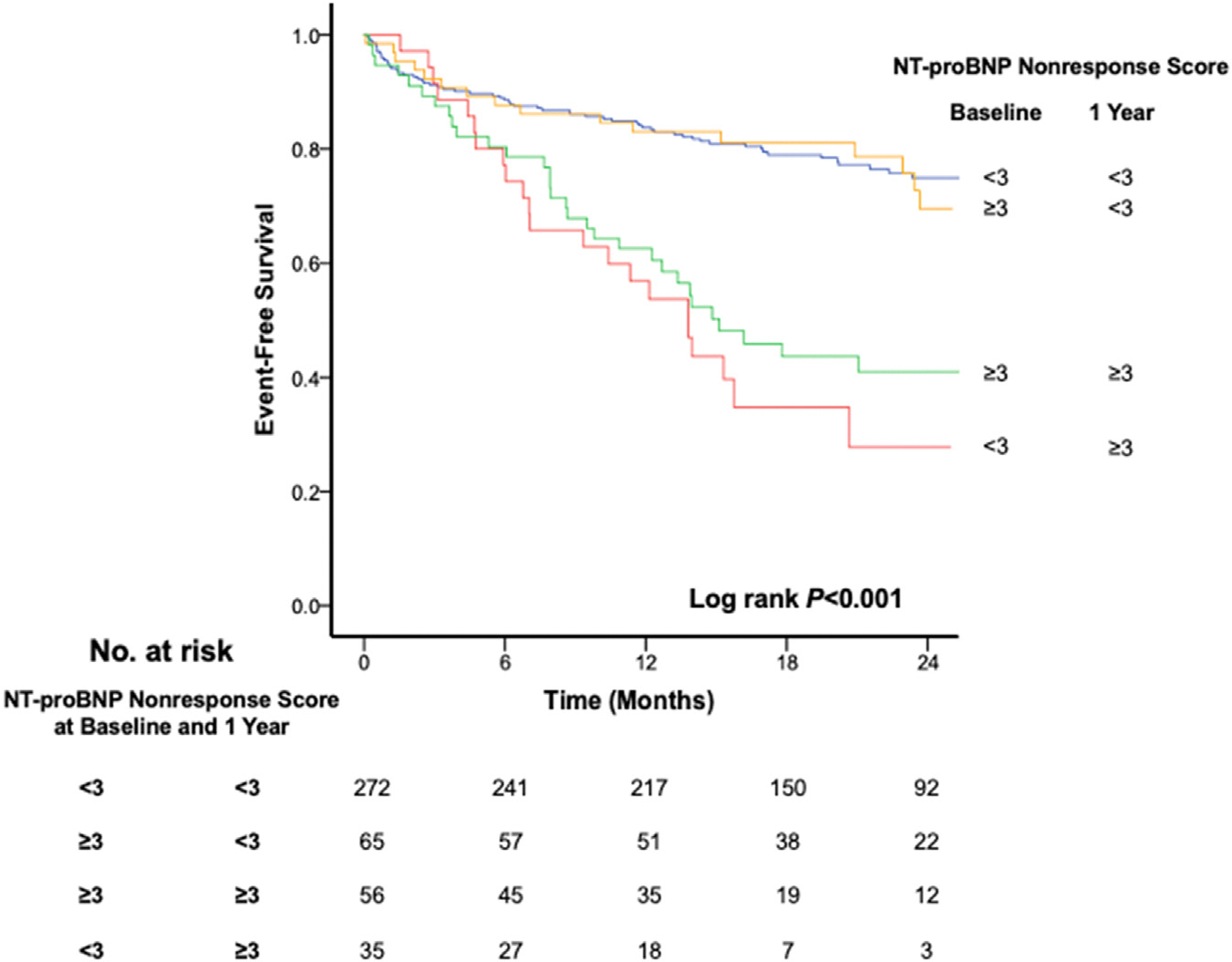
Kaplan-Meier estimates for cardiovascular mortality or heart failure hospitalization in patients with low and high NT-proBNP nonresponse risk score at baseline and 1 year. Patients with low NT-proBNP nonresponse risk score (≤3) at 1 year regardless of their baseline score had significantly improved survival from cardiovascular (CV) mortality and heart failure (HF) hospitalization compared to patients with high score at 1 year. Among patients with high score at 1 year, patients who initially had low score at baseline appeared to have worse prognosis compared to those with persistently high score.

We also explored serial assessment of NNRS in the GUIDE-IT study population. There were 428 patients who were alive at 12 months and had variables available for NNRS calculation. A total of 272 (63.6%) patients had low NNRS at both baseline and 1 year, 35 (8.2%) patients had low NNRS at baseline but high NNRS at 1 year, 65 (15.2%) patients had high NNRS at baseline but low NNRS at 1 year, and 56 (13.1%) patients had high NNRS at both baseline and 1 year. Baseline characteristics across all the groups are shown in Supplementary Table S2. Patients with low NNRS score 1 year had highest survival from CV death and HF hospitalization, regardless of their baseline score, while those with persistently high NNRS or high at 1 year had lowest survival (Fig. 3).

## Discussion

In our present study, first, we observed that baseline NT-proBNP and NNRS slightly outperformed baseline health status measures including KCCQ-OSS, DASI and 6MWD, in identifying NT-proBNP response at 6 and 12 months in patients with HFrEF. Higher NNRS and lower health status measures (especially KCCQ-OSS) were significantly associated with lack of NT-proBNP response at 6 and 12 months, as well as an increased risk of CV mortality and HF hospitalization that was independent of age, sex, co-morbidities, LVEF, baseline creatinine and NT-proBNP levels. Third, NNRS and KCCQ-OSS could be used complimentarily for risk stratification. Fourth, patients who had persistently high and rising to high NNRS at 1 year appeared to have poor prognosis. These findings support the validated role of NNRS (especially in combination with health status measures) to identify patients with HFrEF that need further intensification of drug/device interventions, closer (or even more dedicated physiologic) monitoring, and evaluation for advanced HF therapies (Graphical Abstract). Although prior studies have highlighted the prognostic value of persistently elevated NT-proBNP in chronic stable HF, our report provides a clinically relevant insight in the form of an external validated risk score that can be readily applied at the bedside across a wide clinical spectrum.

The NNRS seemed to stratify patients with chronic HF according to their comorbidity burden and health status with patients in the higher tertiles being older, having more comorbidity burden, symptoms, and lower use of GDMT. However, there appeared to be a statistically significant difference in the LVEF between patients in tertile 1 (21 [18–30]), tertile 2 and 3 (25 [20–30] and 25 [20–31], respectively), but the difference in the median values was 4 with a relatively similar interquartile range. Given the magnitude of difference and that there is technical variability in LVEF measurements on echocardiography, it is unlikely that this difference was clinically or prognostically meaningful.

In the original study,^12^ the C-statistic of the NNRS was 0.82 in the derivation cohort, and 0.73 in the validation cohort,^12^ which appears to be similar to the C-statistic of 0.72 in the GUIDE-IT cohort. The discriminative capacity for NT-proBNP response in 6 to 12 months of the NNRS appeared to slightly outperform all the health status measures, which might be driven mainly by baseline NT-proBNP levels included in NNRS. In the multivariable logistic regression analysis adjusting for age, sex, co-morbidities, LVEF, baseline creatinine and NT-proBNP levels, the NNRS remained marginally predictive for NT-proBNP response at 12 months but not at 6 months. This finding may suggest that achieving target NT-proBNP level early depends mostly on baseline NT-proBNP, whereas the longer trend of NT-proBNP could be affected by multiple factors.^10,19^ Although beta-blockers can result in bradycardia, and, therefore, higher NNRS, but their cardioprotective effect on the sympathetic nervous system may help contribute to lower baseline NT-proBNP, symptom burden, NNRS and a decrease in NT-proBNP over time. The NNRS offers a simple and comprehensive approach to risk stratification by combining baseline NT-proBNP levels, heart rate and symptoms. Furthermore, we validated the prognostic value of the NNRS combined with all the health status measures for CV mortality and HF hospitalization, which remained robust after adjusting for cardiorenal risk factors and baseline NT-proBNP levels. This suggests that the NNRS and health status can be used complimentarily for prognostication in patients with HFrEF. Interestingly, in our analysis on NNRS changes from baseline to 1 year, we found that patients who initially had low score at baseline but later had high score at 1 year appeared to have even worse prognosis that patients with persistently high score. This group of patients may represent patients with relatively more progressive disease with rapid deterioration of their HF than patients with chronically elevated NT-proBNP/risk score. Our findings supported the prognostic value of interval monitoring of natriuretic peptide levels (or its derived risk score) over time may as suggested by the latest HF guidelines.^19^ Another recent *post hoc* analysis of the same GUIDE-IT study also demonstrated that serial NT-proBNP testing, compared to single measurement at baseline, provided significant incremental prognostic value, and improved risk reclassification.^21^ Our findings not only validated prior nonresponse risk score but also described the prevalence of residual risks in a HFrEF population receiving contemporary GDMT, and provided clinically relevant thresholds defined by NNRS for such residual risks that is incremental to patient perceived health status (KCCQ) or functional capacity (DASI and 6MWD).

Natriuretic peptide levels and health status measures have long been recognized to have modest correlations.^22^ Although the KCCQ, DASI and 6MWD have been shown to correlate with severity of HF, natriuretic peptide levels and prognosis in patients with HFrEF,^13–18^ their role in predicting natriuretic peptide response following GDMT titration has not been well established. Lower NT-proBNP levels have long been associated with improved KCCQ scores at 90 days in patients with HFrEF enrolled in the GUIDE-IT trial.^4^ In our present study, we showed that lower baseline KCCQ-OSS was associated with NT-proBNP response at 6 and 12 months independently of baseline NT-proBNP, whereas DASI and 6MWD were not associated with NT-proBNP response after adjusting for baseline NT-proBNP. Currently, KCCQ has been recommended as one of the preferred HF-specific health status assessments that are sensitive to changes in HF severity and responsive to HF therapy including GDMT.^19^ Based on our findings, in addition to NT-proBNP levels, baseline KCCQ may have potential to identify patients who are likely to not respond to GDMT initiation and titration, and who might benefit from more GDMT intensification. Therefore, given these findings and that the NNRS offers a more comprehensive approach to risk stratification, and provides incremental prognostic value compared to NT-proBNP levels, the NNRS may be used complimentarily with KCCQ in routine clinical practice for prognostication in patients with chronic HF who may be at higher risk and benefit from a more intensified GDMT titration and follow up.

## Limitations

There are several limitations that should be considered in the interpretation of our findings. First, dichotomization of the NNRS and health status measures based on the median values was used in the statistical analyses to simplify their potential clinical application. Dichotomization may also increase the change of type I and type II error, as well as significant heterogeneity of the patients within each group. Second, given that the median values were used as the cut-offs for the NNRS and health status measures, standardized cut-offs with external validation remain to be elucidated. Third, correction for multiple comparison testing was not performed, and, therefore, the results should be considered exploratory for further research as there are limited data on the combined use of NT-proBNP, symptoms and health status measures for prognostication in patients with chronic HF. Fourth, the GUIDE-IT trial was conducted prior to the routine clinical use of contemporary GDMT such as angiotensin receptor/neprilysin inhibitors and sodium-glucose cotransporter-2 inhibitors. Fifth, there were patients in the GUIDE-IT cohort that had missing data for NNRS calculation and were excluded from the study, which might contribute to potential selection bias in our analyses. Last, there are other standard HF-specific health status measures that have also been recommended in the current HF guidelines.^19^

## Conclusion

The NNRS and KCCQ may have potential to predict NT-proBNP response at 6 and 12 months independently of cardiorenal risk factors and baseline NT-proBNP levels in patients with HFrEF. In addition, the NNRS, KCCQ-OSS, DASI and 6MWD are associated with prognosis and can be used complimentarily for risk stratification in patients with HFrEF.

## Supplementary Material

1

Supplementary material associated with this article can be found, in the online version, at doi:10.1016/j.ahj.2025.01.011.

## Figures and Tables

**Table 1.** Baseline characteristics across NT-proBNP nonresponse score tertiles

Characteristic	All (*n* = 877)	Tertile 1 (*n* = 314)	Tertile 2 ( *n* = 275)	Tertile 3 (*n* = 288)	*P* value

NT-proBNP nonresponse score range		0–2	3	4–6	
Age (years)	63 (53–71)	59 (49–67)	61 (53–72)	67 (59–75)	< .001
Male, n (%)	596 (68)	202 (64.3)	189 (68.7)	205 (71.2)	.188
Body mass index (kg/m^2^ )	28.8 (24.6–33.9)	29.1 (25.2–35.4)	29.2 (24.6–33.6)	27.5 (24.0–32.7)	.023
Comorbidities					
Ischemic heart disease, n (%)	403 (46)	106 (33.8)	132 (48)	165 (57.3)	< .001
Stroke, n (%)	93 (10.6)	19 (6.1)	29 (10.5)	45 (15.6)	.001
Atrial fibrillation, n (%)	351 (40)	38 (12.1)	104 (37.8)	209 (72.6)	< .001
Hypertension, n (%)	693 (79)	239 (76.1)	212 (77.1)	242 (84)	.037
COPD, n (%)	190 (21.7)	60 (19.1)	56 (20.4)	74 (25.7)	.120
Diabetes mellitus, n (%)	405 (46.2)	121 (38.5)	126 (45.8)	158 (54.9)	< .001
Chronic kidney disease, n (%)	324 (36.9)	67 (21.3)	94 (34.2)	163 (56.6)	< .001
Dyslipidemia, n (%)	513 (58.5)	152 (48.4)	165 (60)	196 (68.1)	< .001
Peripheral artery disease, n (%)	94 (10.7)	21 (6.7)	32 (11.6)	41 (14.2)	.010
Left ventricular ejection fraction (%)	23 (20–30)	21 (18–30)	25 (20–30)	25 (20–31)	.005
NYHA functional class					< .001
I, n (%)	58 (6.6)	42 (13.4)	15 (5.5)	1 (0.3)	
II, n (%)	446 (50.9)	234 (74.5)	157 (57.1)	55 (19.1)	
III, n (%)	356 (40.6)	37 (11.8)	99 (36)	220 (76.4)	
IV, n (%)	17 (1.9)	1 (0.3)	4 (1.5)	12 (4.2)	
Creatinine, mg/dL	1.4 (1.1–2.0)	1.2 (1.0–1.6)	1.4 (1.1–2.1)	1.7 (1.2–2.7)	< .001
Medications					
ACEI/ARB, n (%)	699 (79.7)	280 (89.2)	214 (77.8)	205 (71.2)	< .001
Beta-blocker, n (%)	832 (94.9)	300 (95.5)	260 (94.5)	272 (94.4)	.632
MRA, n (%)	436 (49.7)	169 (53.8)	137 (49.8)	130 (45.1)	.104
Digoxin, n (%)	182 (20.8)	47 (15)	61 (22.2)	74 (25.7)	.004
Loop diuretics, n (%)	838 (95.6)	293 (93.3)	267 (97.1)	278 (96.5)	.053
NT-proBNP, pg/mL					
Baseline	2626 (1468–5202)	1357 (615–2321)	2842 (1885–4384)	5964 (3254–9397)	< .001
3 months	1811 (776–3760)	857 (319–1804)	2057 (1109–3717)	3562 (1806–6487)	< .001
6 months	1572 (613–3621)	737 (205–1661)	1844 (935–4079)	3011 (1337–6094)	< .001
12 months	1231 (422–2858)	598 (157–1490)	1594 (640–3212)	2395 (1161–5199)	< .001
Functional status measures					
KCCQ-OSS	59 (40–75)	65 (46–79)	60 (39–76)	52 (34–68)	< .001
Duke activity status index	7.3 (2.8–15.5)	10.7 (4.5–21.5)	7.3 (2.8–15.5)	7.2 (2.2–10.7)	< .001
6-minute walk distance (meters)	305 (202–375)	332 (240–402)	300 (183–367)	228 (151–318)	< .001
All-cause mortality, n (%)	140 (16)	25 (8)	35 (12.7)	80 (27.8)	< .001
Cardiovascular mortality, n (%)	107 (12.2)	18 (5.7)	27 (9.8)	62 (21.5)	< .001
Heart failure hospitalization, n (%)	282 (32.2)	64 (20.4)	97 (32.3)	121 (42)	< .001

Values are median (IQR). P values are from Kruskall-Wallis or Chi-square test.

Abbreviations: ACEI, angiotensin-converting enzyme inhibitor; ARB, angiotensin receptor blocker; COPD, chronic obstructive pulmonary disease; KCCQ-OSS, Kansas city cardiomyopathy questionnaire overall summary score; MRA, mineralocorticoid receptor antagonist; NT-proBNP, N-terminal pro-B-type natriuretic peptide; NYHA, New York heart association.

**Table 2.** Univariable logistic regression for NT-proBNP nonresponse at 6, 12 months and composite of cardiovascular mortality or heart failure hospitalization

Parameters	NT-proBNP nonresponse at 6 months		NT-proBNP nonresponse at 12 months		Composite of cardiovascular mortality and heart failure hospitalization	
	Odds ratio (95% CI)	*P* value	Odds ratio (95% CI)	*P* value	Odds ratio (95% CI)	*P* value

NNRS ≥3	3.97 (2.56–6.15)	< .001	4.01 (2.47–6.52)	< .001	1.97 (1.58–2.46)	< .001
KCCQ-OSS < median	1.54 (1.10–2.17)	.012	1.68 (1.15–2.47)	.008	1.63 (1.31–2.03)	< .001
Duke activity status index < median	1.51 (1.08–2.12)	.017	1.54 (1.05–2.25)	.026	1.58 (1.27–1.98)	< .001
6-minute walk distance < median	2.24 (1.55–3.23)	< .001	2.64 (1.74–3.99)	< .001	2.10 (1.63–2.70)	< .001

Abbreviations: CI, confidence interval; KCCQ-OSS, Kansas city cardiomyopathy questionnaire overall summary score; NNRS, N-terminal pro-B-type natriuretic peptide nonresponse score; NT-proBNP, N-terminal pro-B-type natriuretic peptide.

**Table 3.** Multivariable logistic regression for NT-proBNP nonresponse at 6, 12 months and composite of cardiovascular mortality or heart failure hospitalization

Parameters	NT-proBNP nonresponse at 6 months		NT-proBNP nonresponse at 12 months		Composite of cardiovascular mortality and heart failure hospitalization	
	Adjusted odds ratio (95% CI)	*P* value	Adjusted odds ratio (95% CI)	*P* value	Adjusted odds ratio (95% CI)	*P* value

NNRS ≥3	1.82 (1.00–3.30)	.050	1.99 (1.02–3.90)	.044	1.79 (1.36–2.36)	< .001
KCCQ-OSS < median	1.55 (1.03–2.32)	.034	2.14 (1.33–3.45)	.002	1.58 (1.25–1.99)	< .001
Duke Activity Status Index < median	1.23 (0.82–1.84)	.319	1.52 (0.96–2.40)	.075	1.49 (1.18–1.88)	.001
6-minute walk distance < median	1.30 (0.80–2.10)	.291	1.59 (0.92–2.75)	.099	1.82 (1.37–2.42)	< .001

Multivariable model: adjusted for age, sex, body mass index, comorbidities (ischemic heart disease, stroke, atrial fibrillation, hypertension, chronic obstructive pulmonary disease, diabetes mellitus, dyslipidemia, and peripheral artery disease), left ventricular ejection fraction, baseline creatinine and NT-proBNP.

Abbreviations: CI, confidence interval; KCCQ-OSS, Kansas city cardiomyopathy questionnaire overall summary score; NNRS, N-terminal pro-B-type natriuretic peptide nonresponse score; NT-proBNP, N-terminal pro-B-type natriuretic peptide.

## Abbreviation

6MWD: 6-minute Walk Distance
AF: Atrial Fibrillation
BMI: Body Mass Index
CV: Cardiovascular
DASI: Duke Activity Status Index
GDMT: Guideline Directed Medical Therapy
GUIDE-IT: The Guiding Evidence Based Therapy Using Biomarker Intensified Treatment in Heart Failure
HF: Heart Failure
HFrEF: Heart Failure with Reduced Ejection Fraction
KCCQ: Kansas City Cardiomyopathy Questionnaire
LVEF: Left Ventricular Ejection Fraction
NNRS: NT-proBNP Non-Response Score
NT-proBNP: N-terminal Pro-B-type Natriuretic Peptide
NYHA: New York Heart Association
