# Supplementary material for: Baseline NT-proBNP nonresponse score and health status measures in assessing treatment responses in heart failure with reduced ejection fraction
Source: Am Heart J. Author manuscript; Available in PMC 2026 May 30. (PMC13221967; doi:10.1016/j.ahj.2025.01.011)
Supplement: 1 [file NIHMS2170126-supplement-1.docx]

**SUPPLEMENTAL MATERIAL**

**Usefulness of Baseline NT-proBNP Non-Response Score and Health Status Measures in Assessing Treatment Responses: Validation in the GUIDE-IT Trial.**

Thanat Chaikijurajai MD^1^, Horng H. Chen MBBCh,^1^ W. H. Wilson Tang MD^2^

^1^Department of Cardiovascular Medicine, Mayo Clinic, Rochester MN

^2^Kaufman Center for Heart Failure Treatment and Recovery, Heart Vascular and Thoracic Institute, Cleveland Clinic, Cleveland OH.

**Supplemental Figure S1:** Scatter Plots with Median Values and Spearman’s Correlation Coefficients Among and Health Status Measures

**
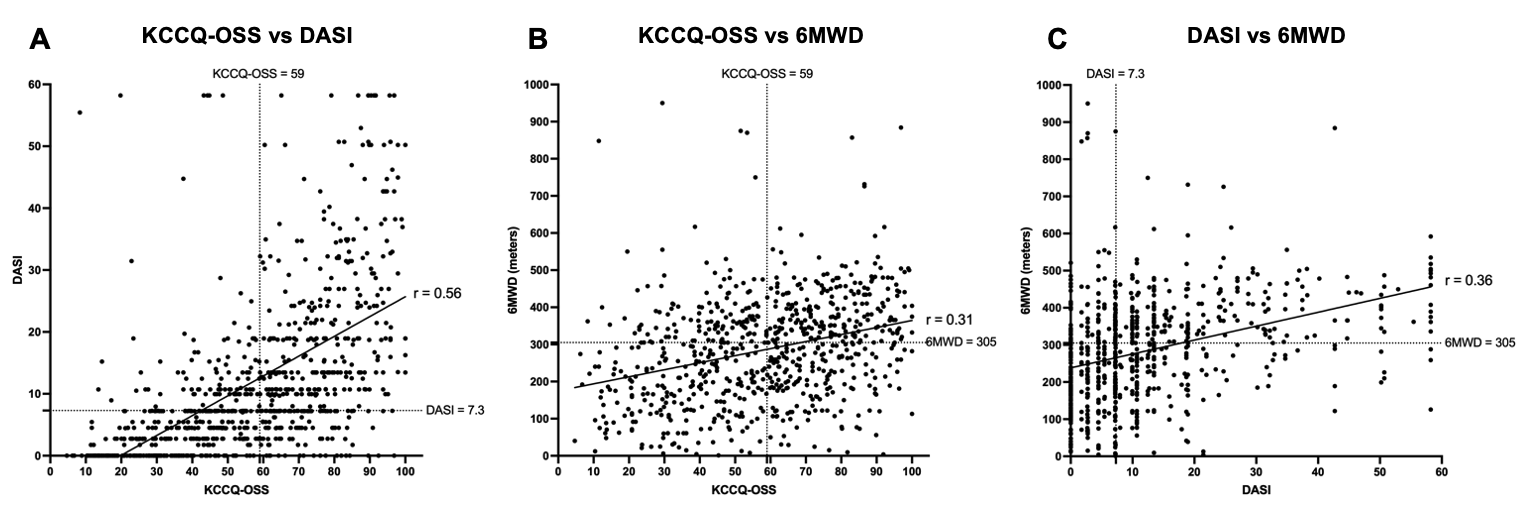
**

Caption: Kansas City Cardiomyopathy Questionnaire overall summary score (KCCQ-OSS, median value 59) and Duke Activity Status Index (DASI, median value 7.3) correlated modestly with Spearman’s Correlation Coefficients (r) of 0.56 (**A**) but correlated poorly with 6-minute walk distance (6MWD, median value 305 meters) with r of 0.31 (**B**). DASI and 6MWD also correlated poorly with r of 0.36 (**C**).

**Supplemental Figure S2:** Kaplan-Meier Estimates for Cardiovascular Mortality or Heart Failure Hospitalization Across NT-proBNP Nonresponse Score Tertiles.


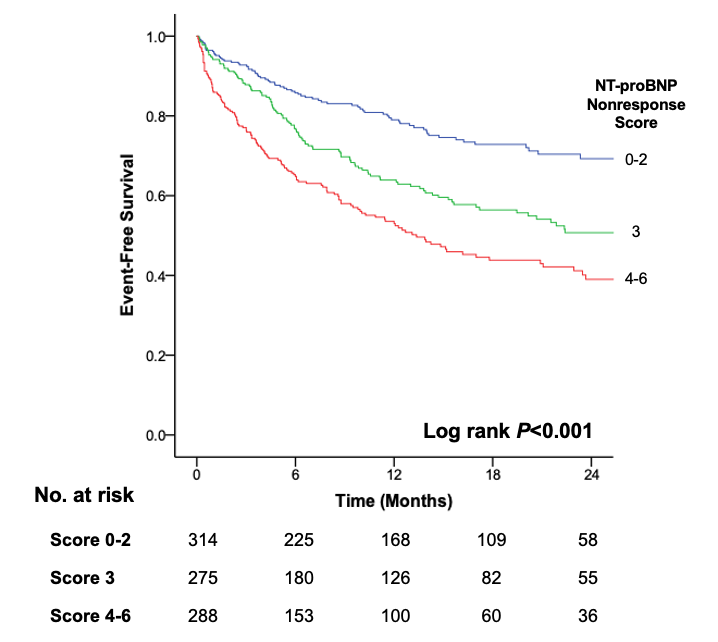


Patients with the NT-proBNP nonresponse score in the middle tertile (score 3) and highest tertile (score 4-6) had worse survival from cardiovascular (CV) mortality and heart failure (HF) compared to patients with the NT-proBNP nonresponse score in the first tertile (score 0-2).

**Supplemental Table S1**: C-statistics of NT-proBNP Nonresponse Score, Baseline NT-proBNP and Health Status Measures for NT-proBNP Response at 6 Months, 12 Months and Composite of Cardiovascular Mortality or Heart Failure Hospitalization

| **Parameters** | **NT-proBNP Nonresponse at 6 Months** | | **NT-proBNP Nonresponse at 12 Months** | | **Composite of Cardiovascular Mortality or Heart Failure Hospitalization** | |
| --- | --- | --- | --- | --- | --- | --- |
|  | **C-statistic (95% CI)** | **P Value** | **C-statistic (95% CI)** | **P value** | **C-statistic (95% CI)** | **P value** |
| Baseline NT-proBNP levels | 0.78 (0.74-0.82) | <0.001 | 0.75 (0.70-0.80) | <0.001 | 0.60 (0.57-0.64) | <0.001 |
| NNRS | 0.73 (0.69-0.78) | <0.001 | 0.72 (0.67-0.77) | <0.001 | 0.64 (0.60-0.67) | <0.001 |
| KCCQ-OSS | 0.56 (0.51-0.60) | 0.026 | 0.59 (0.54-0.65) | 0.001 | 0.60 (0.56-0.64) | <0.001 |
| Duke Activity Status Index | 0.54 (0.50-0.59) | 0.080 | 0.56 (0.51-0.62) | 0.029 | 0.57 (0.53-0.60) | 0.001 |
| 6-minute walk distance | 0.63 (0.58-0.67) | <0.001 | 0.65 (0.60-0.70) | <0.001 | 0.62 (0.58-0.66) | <0.001 |

Abbreviation: CI = confidence interval; KCCQ-OSS = Kansas City Cardiomyopathy Questionnaire Overall Summary Score; NNRS = N-terminal pro-B-type natriuretic peptide nonresponse score; NT-proBNP = N-terminal pro-B-type natriuretic peptide

**Supplemental Table S2**: Baseline Characteristics across Patients with low/high NT-proBNP Nonresponse Score at Baseline and 1 Year

| **Characteristic** | **All (n=428)** | **Low/Low (n=272)** | **Low/High (n=35)** | **High/Low (n=65)** | **High/High (n=56)** | **P Value** |
| --- | --- | --- | --- | --- | --- | --- |
| NT-proBNP nonresponse score at baseline and 1 year |  | ≤3 and ≤3 | ≤3 and >3 | >3 and ≤3 | >3 and >3 |  |
| Age (years) | 63 (52-70) | 58 (50-68) | 66 (62-71) | 65 (56-69) | 68 (63-74) | <0.001 |
| Male, n(%) | 292 (68.2) | 179 (65.8) | 25 (71.4) | 48 (73.8) | 40 (71.4) | 0.550 |
| Body mass index (kg/m^2^) | 28.8 (24.8-33.7) | 29.2 (25.0-34.6) | 29.4 (25.0-35.3) | 26.5 (23.0-31.8) | 28.0 (25.7-32.7) | 0.165 |
| Comorbidities | | | | | | |
| Ischemic heart disease, n(%) | 184 (43.0) | 103 (37.9) | 17 (48.6) | 31 (47.7) | 33 (58.9) | 0.021 |
| Stroke, n(%) | 41 (9.6) | 21 (7.7) | 4 (11.4) | 8 (12.3) | 8 (14.3) | 0.360 |
| Atrial fibrillation, n(%) | 175 (40.9) | 64 (23.5) | 19 (54.3) | 44 (67.7) | 48 (85.7) | <0.001 |
| Hypertension, n(%) | 331 (77.3) | 198 (72.8) | 29 (82.9) | 57 (87.7) | 47 (83.9) | 0.027 |
| COPD, n(%) | 82 (19.2) | 46 (16.9) | 8 (22.9) | 17 (26.2) | 11 (19.6) | 0.354 |
| Diabetes mellitus, n(%) | 193 (45.1) | 110 (40.4) | 18 (51.4) | 38 (58.5) | 27 (48.2) | 0.049 |
| Chronic kidney disease, n(%) | 142 (33.2) | 55 (20.2) | 21 (60.0) | 30 (46.2) | 36 (64.3) | <0.001 |
| Dyslipidemia, n(%) | 244 (57.0) | 139 (51.1) | 23 (65.7) | 39 (60.0) | 43 (76.8) | 0.003 |
| Peripheral artery disease, n(%) | 46 (10.7) | 24 (8.8) | 7 (20.0) | 7 (10.8) | 8 (14.3) | 0.179 |
| Left ventricular ejection fraction (%) | 23 (19-30) | 21 (17-29) | 25 (20-33) | 25 (15-33) | 25 (20-30) | 0.006 |
| NYHA Functional Class | | | | | | <0.001 |
| I, n(%) | 35 (8.2) | 33 (12.1) | 1 (2.9) | 1 (1.5) | 0 (0.0) |  |
| II, n(%) | 230 (53.7) | 177 (65.1) | 28 (80.0) | 14 (21.5) | 11 (19.6) |  |
| III, n(%) | 157 (36.7) | 58 (21.3) | 6 (17.1) | 49 (75.4) | 44 (78.6) |  |
| IV, n(%) | 6 (1.4) | 4 (1.5) | 0 (0.0) | 1 (1.5) | 1 (1.8) |  |
| Creatinine, mg/dL | 1.30 (1.05-1.85) | 1.21 (0.99-1.74) | 1.68 (1.14-2.28) | 1.40 (1.14-2.00) | 1.72 (1.35-2.94) | <0.001 |
| Medications | | | | | | |
| ACEI/ARB, n(%) | 367 (84.3) | 240 (88.2) | 27 (77.1) | 54 (83.1) | 40 (71.4) | 0.009 |
| Beta-blocker, n(%) | 411 (96.0) | 261 (96.0) | 33 (94.3) | 64 (98.5) | 53 (94.6) | 0.665 |
| MRA, n(%) | 211 (49.3) | 141 (51.8) | 17 (48.6) | 31 (47.7) | 22 (39.3) | 0.388 |
| Digoxin, n(%) | 101 (23.6) | 55 (20.2) | 10 (28.6) | 16 (24.6) | 20 (35.7) | 0.079 |
| Loop diuretics, n(%) | 410 (95.8) | 260 (95.6) | 32 (91.4) | 63 (96.9) | 55 (98.2) | 0.440 |
| NT-proBNP, pg/mL | | | | | | |
| Baseline | 2138 (1351-4288) | 1796 (968-3173) | 2931 (2315-4014) | 4331 (2029-7241) | 5527 (3526-8806) | <0.001 |
| 3 months | 1614 (713-3223) | 1175 (451-2043) | 3753 (2484-4809) | 1789 (1349-3600) | 3939 (2705-7135) | <0.001 |
| 6 months | 1272 (457-2498) | 904 (317-1946) | 4181 (1918-5970) | 1251 (763-2441) | 4039 (2201-7733) | <0.001 |
| 12 months | 1231 (415-2858) | 758 (220-1713) | 6336 (3019-12830) | 1150 (620-2449) | 4129 (2243-6906) | <0.001 |
| Functional Status Measures | | | | | | |
| KCCQ-OSS | 61 (42-78) | 63 (45-79) | 63 (34-78) | 59 (48-78) | 47 (38-64) | 0.003 |
| Duke Activity Status Index | 8 (4.5-15.2) | 10 (4.5-18.3) | 7.2 (2.7-10.7) | 10 (5.5-14.3) | 7.2 (2.8-10.7) | 0.016 |
| 6-minute walk distance (meters) | 305 (199-373) | 330 (221-396) | 263 (174-338) | 257 (194-361) | 225 (122-305) | <0.001 |
| All-cause mortality, n(%) | 33 (7.7) | 11 (4.0) | 6 (17.1) | 2 (3.1) | 14 (25.0) | <0.001 |
| CV mortality, n(%) | 24 (5.6) | 7 (2.6) | 5 (14.3) | 1 (1.5) | 11 (19.6) | <0.001 |
| HF hospitalization, n(%) | 124 (29.0) | 59 (21.7) | 21 (60.0) | 16 (24.6) | 28 (50.0) | <0.001 |

Values are median (IQR). P values are from Kruskall-Wallis or Chi-square test.

Abbreviation: ACEI = angiotensin-converting enzyme inhibitor; ARB = angiotensin receptor blocker; COPD = chronic obstructive pulmonary disease; KCCQ-OSS = Kansas City Cardiomyopathy Questionnaire Overall Summary Score; MRA = mineralocorticoid receptor antagonist; NT-proBNP = N-terminal pro-B-type natriuretic peptide; NYHA = New York Heart Association.
